# Effects of Local and Systemic Immune Challenges on the Expression of Selected Salivary Genes in the Malaria Mosquito *Anopheles coluzzii*

**DOI:** 10.3390/pathogens10101300

**Published:** 2021-10-09

**Authors:** Giulia Bevivino, Bruno Arcà, Fabrizio Lombardo

**Affiliations:** Department of Public Health and Infectious Diseases, Sapienza University of Rome, P.le Aldo Moro, 5-00185 Rome, Italy; giulia.bevivino@uniroma1.it (G.B.); bruno.arca@uniroma1.it (B.A.)

**Keywords:** *Anopheles coluzzii*, salivary glands, antimicrobial peptides, feeding behaviour, innate immunity, transmission

## Abstract

Salivary glands play a crucial tripartite role in mosquito physiology. First, they secrete factors that greatly facilitate both sugar and blood meal acquisition. Second, the transmission of pathogens (parasites, bacteria and viruses) to the vertebrate host requires both the recognition and invasion of the salivary glands. Third, they produce immune factors that both protect the organ from invading pathogens and are also able to exert their activity in the crop and the midgut when saliva is re-ingested during feeding. Studies on mosquito sialomes have revealed the presence of several female and/or male salivary gland-specific or enriched genes whose function is completely unknown so far. We focused our attention on these orphan genes, and we selected, according to sequence and structural features, a shortlist of 11 candidates with potential antimicrobial properties. Afterwards, using qPCR, we investigated their expression profile at 5 and 24 h after an infectious sugar meal (local challenge) or thoracic microinjection (systemic challenge) of Gram-negative (*Escherichia coli*, EC) or Gram-positive (*Staphylococcus aureus*, SA) bacteria. We observed a general increase in the transcript abundance of our salivary candidates between 5 and 24 h after local challenge. Moreover, transcriptional modulation was determined by the nature of the stimulus, with salivary gland-enriched genes (especially *hyp15* upon SA stimulus) upregulated shortly after the local challenge and later after the systemic challenge. Overall, this work provides one of the first contributions to the understanding of the immune role of mosquito salivary glands. Further characterization of salivary candidates whose expression is modulated by immune challenge may help in the identification of possible novel antimicrobial peptides.

## 1. Introduction

Mosquito females need a blood meal to attain nutrients for egg development. A successful blood meal takes advantage of on the activity of anti-hemostatic factors in the mosquito saliva that may also act as carriers of several pathogens transmitted by mosquitoes, such as parasites of the genus *Plasmodium* [1]. Female mosquitoes initially probe under the host’s skin and salivate, searching for a blood vessel to pierce and to start sucking blood from. This process exposes mosquito mouthparts to microbes on the skin surface of the host that might contaminate the ingested blood meal. Moreover, both male and female mosquitoes need plant sugar to survive and obtain energy for flying and to enhance reproduction: these food sources are usually stored for several hours in the crop, a gut diverticulum located in the thorax, and could be similarly contaminated by microbes living on nectar and honeydew. Portions of the sugar meal are then moved from the crop to the gut for successive digestive processing [2]. Mosquito saliva is known to carry digestive enzymes such as α-glucosidase, maltase, and amylase [3] and antimicrobial activities, such as lysozyme [4,5,6,7]. These compounds may act inside the crop, where digestive enzymes can start the digestion of nutrients taken with the sugar meal, while antimicrobial factors can protect the mosquito from the establishment and dissemination of microbes [2,8]. In fact, it is well known that part of the saliva secreted during the acquisition of the sugar meal reaches first the crop and is then regurgitated and re-directed into the midgut through intestinal peristaltic movements. This physiological process provides an early protection against pathogenic microorganisms, even in the anterior region of the gut [9].

Mosquito salivary glands also play an essential role in the transmission of pathogens such as *plasmodium* parasites and arboviruses (i.e., arthropod-borne viruses, as, for instance, the Zika, dengue, yellow fever, and chikungunya viruses). Indeed, these pathogens need to invade the salivary glands and reach the salivary duct to be transmitted to vertebrate hosts during a blood meal. The process of salivary gland invasion is mediated by molecular interactions between the receptors on the surface of the salivary glands and the ligands on the pathogens [6,10,11]. Interestingly, diverse studies conducted in *Anopheles* mosquitoes have shown that salivary glands are able to mount a defence mechanism to limit *Plasmodium* sporozoite invasion. In this context, transcriptional changes of immune-related genes were observed in *Anopheles* salivary glands upon *Plasmodium* invasion, further suggesting the immune properties of this organ [10]. Innate immunity signaling pathways (Toll, IMD, and JNK pathways) are activated in this organ in response to the invasion of dengue, Zika, and chikungunya viruses in *Ae. aegypti* [11]. Moreover, RNA-seq studies have highlighted a modulation of expression of immune genes after the infection of *An. coluzzii* with the murine malaria parasite *Plasmodium berghei*: specifically, clip-domain serine proteases, PRR (pattern recognition receptors), melanization factors, LRR (leucine-rich repeat) proteins, and Imd pathway mediators were indeed the most highly upregulated in the salivary glands [12]. However, there is a dearth of knowledge on salivary gland immunity and their potential role in modulating pathogen transmission needs that requires further investigation.

In the last twenty years, the application of molecular and biochemical techniques has been of great help in the discovery of salivary gland components and in the identification of new activities in the saliva of hematophagous arthropods. The first salivary factors discovered in mosquitoes were involved in the evasion of host hemostatic responses (e.g., platelet inhibitors as the apyrase or vasodilators as the sialokinins) and in the digestion of the sugar meal (enzymes such as amylases and maltases) [3,13]. In the malaria mosquito *An. coluzzii*, both the construction of cDNA libraries and the advent of large-scale transcriptomic and proteomic approaches have greatly improved our knowledge of the salivary composition, leading to the identification of genes specifically expressed in the female mosquito salivary glands, such as members of the D7 family, apyrase, and many others [14,15]. This has allowed for the assembly of a catalogue that includes 71 genes encoding for putative salivary secretory proteins, with 34 showing a specific and/or enriched expression in female salivary glands (therefore encoding for factors potentially involved in blood meal intake) and another 13 expressed in both male and female salivary glands (likely encoding for factors involved in sugar meal intake). Interestingly, only a small fraction of these predicted salivary proteins has been functionally characterized so far [3], with approximately 40% of them showing no sequence similarities to any known protein in various databases [16]. It is therefore expected that some of these salivary orphan genes may encode for the antimicrobial activities involved in the protection of the mosquito against microbial pathogen proliferation after the blood and/or sugar meal.

The insect immune system is composed of diversified and complex levels of defenses against invading pathogens. The first level is certainly represented by multiple physical barriers to pathogens invasion (cuticle and/or epithelial tissues of midgut, salivary glands, and trachea). However, most insect immune defenses act through cellular and humoral responses such as phagocytosis, melanization, and encapsulation, which are mediated by hemocytes and fat body cells that are activated throughout the proteolytic cascades when the physical barrier alone is not able to block the ongoing infection, allowing pathogens to spread rapidly to other organs [17,18]. Inside fat body cells and hemocytes, antimicrobial peptides (AMPs), potent effectors of the innate immunity, are synthetized and secreted into the hemolymph in response to an immune challenge [19,20]. AMP expression is finely tuned by multiple immune-signaling pathways including the Imd, Toll, and JAK-STAT, whose membrane receptors are adapted to recognize different invading organisms such as Gram-positive and Gram-negative bacteria, yeasts, fungi, and viruses. In response to any exogenous exposure that breaches or damages a local barrier, the immune signaling pathways are rapidly activated at both the local (tissue/organ) and systemic (hemocoel) levels to clear the infection using the production of AMPs via signal transduction cascades [21]. Typically, insect AMPs such as Cecropin and Defensin are detectable from 2–6 h until 24–36 h (first and second line of defense, respectively) after an immune stimulation, providing a prompt immune defense against pathogens [22,23,24,25,26]. The evidence of local and systemic immune responses in insects as well as the existence of a molecular communication between different immune organs/tissues is well documented in *Drosophila* and has also recently been described in *Anopheles* mosquitoes. De et al. have shown that tissues can have a synergistic ability to manage a local (endogenous) and systemic (exogenous) infection after microbial exposure in the mosquito *An. stephensi* upon bacterial feeding and bacterial microinjection, respectively [26]. Local immune defenses generally rely on both the physical barrier and on the activation of the immune signaling pathways (e.g., Imd pathway) in the tissues threatened by a bacterial infection, thus producing a compartmental response. Systemic defenses are mainly based on the activation of the Toll and Imd pathways in the fat body and hemocytes where AMPs are produced and secreted into the insect hemolymph, generating a wide immune response [21]. For instance, it was shown that an oral supplement of bacteria (local challenge) significantly modulates AMP expression in the midgut (MG) as well as in the fat body (FB) of *Ae. aegypti* [27] and that a thoracic injury with bacteria (systemic challenge) causes an alteration in the expression of important modulators of antimicrobial immune responses in *An. gambiae* [28].

**Figure 1:**
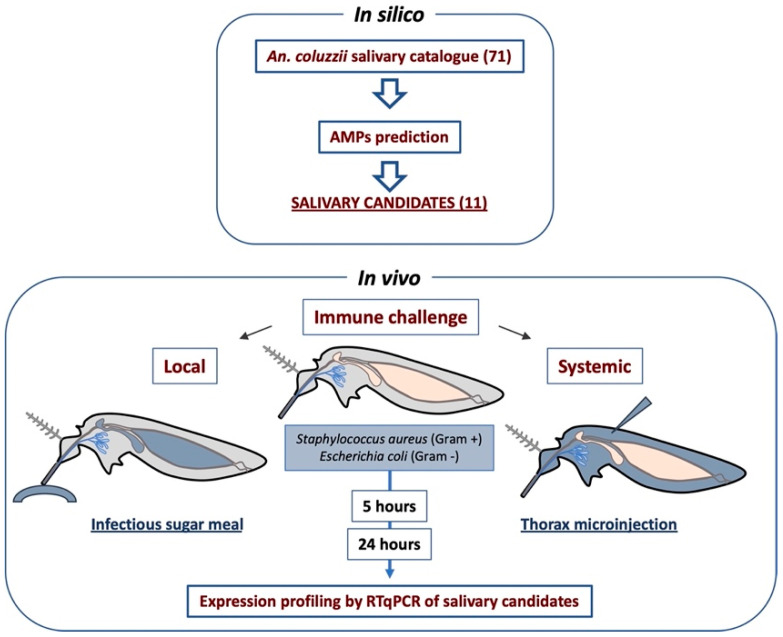
Experimental workflow and technical design. The scheme summarizes the experimental steps of this work. The blue staining in the mosquito drawings corresponds to the tissue mostly involved in the local (crop, midgut) and the systemic (hemocoel) challenges.

The main aim of this work is the identification and initial characterization of putative antimicrobial peptides from the salivary glands of the malaria vector *An. coluzzii*. To this end, the salivary genes encoding putative AMPs were initially selected from the *An. coluzzii* sialome, and the expression profiles (female SG-specific and male/female SG-specific) and structural features (length, amino acid composition, predicted secondary structure) were considered. Among these genes, we included cecropin and salivary lysozyme which are known to act as antimicrobial peptides in insects, as positive controls. We next examined the transcriptional modulation of our selected putative AMPs genes upon a local immune challenge (oral supplement of Gram-positive/Gram-negative bacteria) and a systemic immune challenge (thoracic injection of the same Gram-positive/Gram-negative bacteria strains) at different time-points using the RT-qPCR approach (Figure 1).

## 2. Results

### 2.1. Salivary Candidate Selection

So far, the antimicrobial factors specific to mosquito salivary glands have been poorly described in the literature. It is however plausible that salivary components that are specifically expressed or enriched in the mosquito salivary glands exert such immune activity when sugar and/or blood meals are ingested and/or when pathogens invade the tissue [3]. Using a catalogue of putative *An. gambiae* salivary proteins that was previously compiled [16,29], we selected, on the basis of sequence and structural features, a short list of candidates resembling antimicrobial factors (Figure 1). In more detail, candidate selection was performed according to the following criteria: first, genes with known function or sequence similarity to known proteins (e.g., D7 family members, enzymes as apyrase or maltase, Antigen 5, and SG1 family members) or that were known for their involvement in blood feeding, such as gSG7, gSG7-2, and 30 kDa [30], were excluded from the original catalogue, which included 71 salivary gland genes [16,29]. This filtering generated a catalogue of 26 candidates, including genes with unknown functions and no significant sequence similarity to known proteins according to BLAST analysis using non-redundant databases. These candidates showed four different expression profiles, as previously determined [16,29]: (i) SG, specifically found in female salivary glands; (ii) SG+, expressed abundantly in female salivary glands and weakly in other female and male tissues; (iii) SG F/M, expressed in both male and female salivary glands; and (iv) U (ubiquitous), expressed in salivary glands of both sexes but also in other tissues [31]. From this initial list, we selected those hypothetical proteins with SG, SG+, or SG F/M expression that carried some essential sequence/structural features (short length, hydrophobic profile, basic and/or acidic patterns, presence of a single alpha helix and/or beta-sheet [19]). This selection allowed us to narrow our focus to eight candidates (see Table 1). Salivary genes with the U expression pattern were also screened for their potential involvement in innate immunity by taking advantage of the VectorBase Expression Databases (which include a wide Microarray and RNA-seq data collection). This way, three additional candidate genes (hyp13, hyp14.5 and hyp14.5-1) that are up-regulated after *P. berghei* invasion of mosquito midgut were also selected [32]. To this list of 11 candidates (3 SG+, 5 SG F/M, 3 U), we added the salivary lysozyme and cecropin as controls [5,16] (Table 1).

### 2.2. Molecular Features and Prediction Analyses

Amino acid length, predicted molecular weight, and the isoelectric point of the 11 candidates plus lysozyme and cecropin, with or without the signal peptides, are reported in Table 1. Moreover, the presence of possible secondary proteolytic cleavage sites, which produce mature and active AMPs from a putative immature precursor (pro-peptide), was also predicted using the ProP 1.0 and Spider P tools. Out of 11 candidates, 5 were predicted to need further cleavage processing to produce a functional peptide with possible full activity (Table 1).

The peptide sequences of the precursor and mature salivary candidates (when predicted) were also analyzed using CAMPR3-RF (http://www.camp.bicnirrh.res.in) (accessed on 10 June 2021); this tool allows the probability of a peptide to exert antimicrobial activity to be evaluated, and, according to M. Gabere and W. Noble, provides the best performance among ten AMP prediction servers that are freely available online [33,34]. The results obtained by applying four different algorithms are reported in Table 2.

According to this prediction software, most candidates carried sequence signatures that were typical of antimicrobial peptides, and the probability scores coherent among the different algorithms that were used. Three candidates (hyp14.5, hyp14.5-1, and the mature form of hyp55.3, other than our positive controls, cecropin and salivary lysozyme) showed the highest probability according to all four algorithms. Other candidates were considered as potential antimicrobial peptides according to three (sg2 and sg2a), two (hyp10) or only one (hyp13, hyp12, hyp15 and hyp6.2) out of the four algorithms. Among all the candidates, only hyp17 showed a probability value < 30%. Interestingly, the highest probability of having antimicrobial activity was found among the candidates predicted to undergo proteolytic maturation cleavage.

The propensity of the selected candidates to adopt a different secondary structure (alpha-helix, beta-sheet, coiled coil) was predicted by employing the online tool PSIPRED. Simple sequence and structural features were predicted for the short SG-enriched and the SG-F/M candidates (Figures S1–S3).

### 2.3. Transcriptional Profiling by Local and Systemic Bacterial Challenges

Robust evidence of the occurrence of molecular communication between different immune tissues have been obtained in *Drosophila* in the last twenty years [35,36]. On the contrary, only a few studies are available in mosquitoes, and the existence of a *local* and a *systemic* immune response is only partially understood [26]. However, there is evidence that the oral supplementation of bacteria could significantly alter AMP expression in the MG as well as in the FB of the mosquito *Aedes aegypti* [27]. To understand the regulation of selected salivary genes with a potential role in their interactions with microorganisms, we investigated the transcriptional modulation of salivary candidates upon local and systemic bacterial challenges by quantitative real-time PCR (RTqPCR). Adult *An. coluzzii* females were immune challenged by (i) the administration of a sugar meal infected by Gram-negative *Escherichia coli* (EC) or Gram-positive *Staphylococcus aureus* (SA) or by (ii) thoracic injection of the same bacterial strains. The expression profile of the candidate genes after these immune stimulations (infectious sugar meal or infectious injection) was investigated at 5 h and 24 h post-infection.

#### 2.3.1. Feeding Assays Reveal an Early Transcriptional Increase of SG+ Candidates

**Figure 2:**
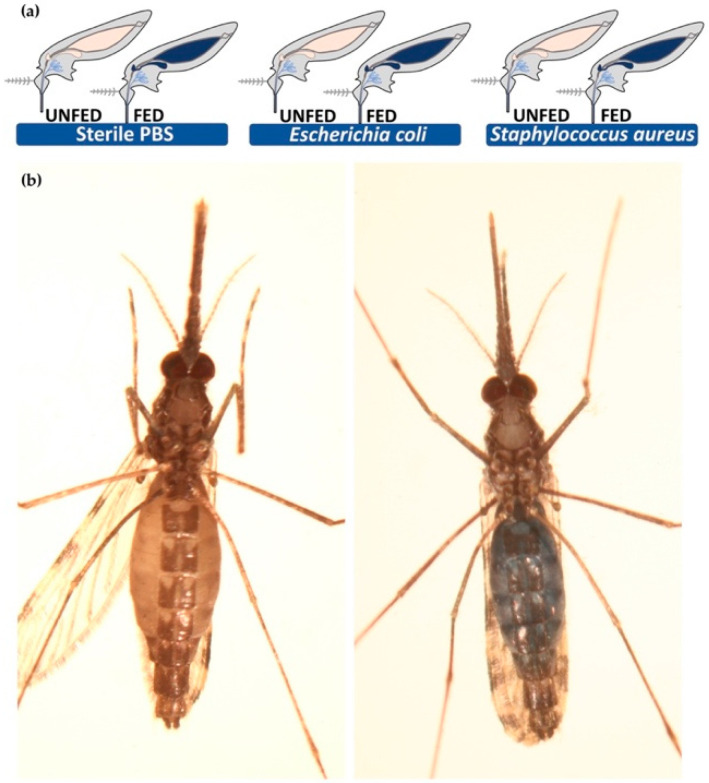
Feeding assay: (**a**) infectious sugar meals were offered to starved mosquitoes as schematically summarized; (**b**) images of sugar fed (right image) and not fed (left image) mosquitoes.

Transcriptional modulation of selected salivary candidates upon *local* immune challenge was investigated by feeding mosquitoes with infectious sugar meals. Five and twenty-four hours were selected as time points that were indicative of the early organ response and of a long-term response to infection, respectively. The stained sugar meal allowed us to easily and effectively select only fed mosquitoes for further analysis (Figure 2).

Three independent experiments were performed using a Gram-negative (EC) or a Gram-positive (SA) strain to elicit the immune response and sterile PBS as a control. The titer of the bacteria used for the infectious meals was defined both according to literature data [26] and to preliminary experiments completed in our lab that aimed to establish a sub-lethal dose of bacteria. 

**Figure 3:**
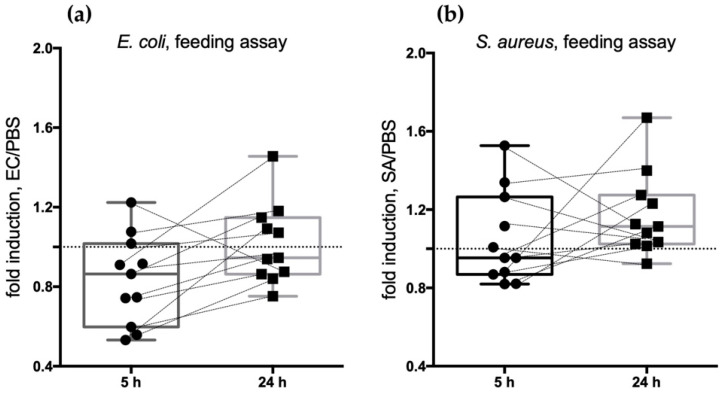
Feeding assay: (**a**) median value of the relative expression (fold induction as ratio between EC-treated and PBS-treated mosquitoes) of each candidate at 5 and 24 h (dots and squares, respectively) after challenge; (**b**) median value of the relative expression (fold induction as ratio between SA-treated and PBS-treated mosquitoes) of each candidate at 5 and 24 h (dots and squares, respectively) after challenge.

Overall, we found that shortly after the challenge (5 h), the transcript abundance of most of our 11 candidates showed a decreasing trend compared to the challenge with PBS, which was used as a reference (Figure 3). This was slightly more evident upon the EC challenge (abundance of 8/11 transcripts reduced) than after the SA challenge (6/11 transcripts). The situation appeared different at 24 h post-challenge, and as a general trend, we could observe an increase in transcript abundance in comparison to sterile PBS-fed mosquitoes (Figure 3). More specifically, expression profile analysis revealed that most of the candidates (10/11) underwent an increase in transcript abundance between 5 and 24 h after the immune challenge with the Gram-negative *E. coli* and, to a lesser extent, after a sugar meal infected with the Gram-positive *S. aureus* (7/11). 

**Figure 4:**
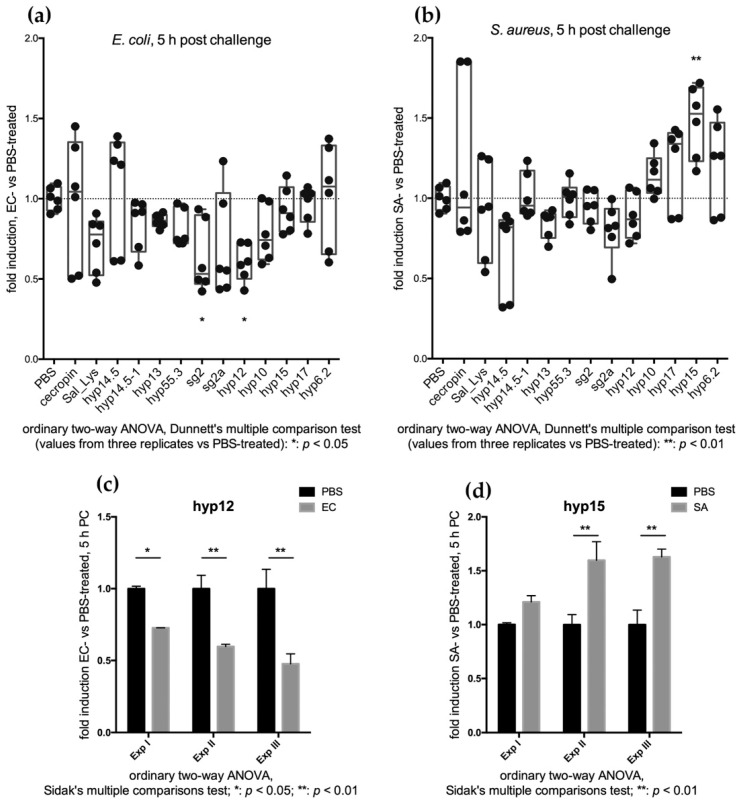
Feeding assay: (**a**) transcriptional variations 5 h after EC infectious sugar meal; (**b**) Transcriptional variations 5 h after SA infectious sugar meal. Biological and technical replicates are reported as dots corresponding to fold induction (ratio between bacteria-treated and PBS-treated mosquitoes). Statistical analysis (2-way ANOVA followed by Dunnett’s multiple comparison test) was applied on transcript fold change values (ratio between bacteria challenged values and sterile PBS challenged values) and is reported below each graph. (**c**) Transcriptional variations of hyp12 5 h after EC infectious sugar meal in the three replicates (2-way ANOVA followed by Sidak’s multiple comparison tests was applied to statistically analyse transcript fold change values). (**d**) Transcriptional variations of hyp15 5 h after SA infectious sugar meal in the three replicates (2-way ANOVA followed by Sidak’s multiple comparison tests was applied to statistically analyse transcript fold change values).

The change in the transcript abundance of each single candidate at 5 h post challenge was further analysed. A significant decrease in the transcript abundance (*p* < 0.05) was only achieved for hyp12 and sg2 upon EC infection (Figure 4a,c). Interestingly, the transcript abundance of the three SG+ candidate genes (*hyp17*, *hyp15* and *hyp6.2*) appeared to increase rather than decrease upon SA infection, which suggests their possible involvement in early local immune response to Gram-positive bacterial challenge (Figure 4); notably, this increase was statistically significant for hyp15 (Figure 4b,d).

**Figure 5:**
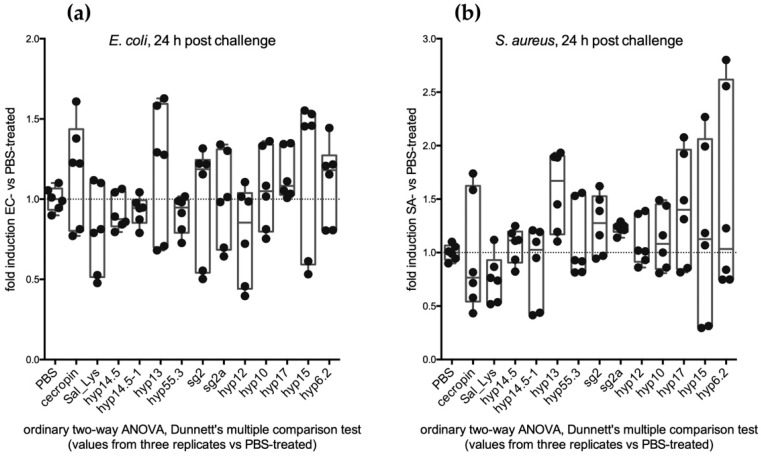
Feeding assay: (**a**) transcriptional variations 24 h after EC infectious sugar meal; (**b**) Transcriptional variations 24 h after SA infectious sugar meal. Biological and technical replicates are reported as dots corresponding to fold induction (ratio between bacteria-treated and PBS-treated mosquitoes). Statistical analysis (2-way ANOVA followed by Dunnett’s multiple comparison test) was applied on transcript fold change values (ratio between bacteria challenged values and sterile PBS challenged values) and is reported below each graph.

At 24 h post-challenge, we could observe a general increase in transcript abundance in comparison to sterile PBS-fed mosquitoes (Figure 3 and Figure 5). This was especially clear when observing the SA-challenged mosquitoes (increased abundance of 10/11 transcripts) compared to the EC-challenged ones (6/11 transcripts), perhaps because, as previously reported, Gram-positive bacteria seem to represent a stronger immune stimulus [37]. This transcriptional increase may indicate that most of the selected genes are somehow part of a physiological mosquito response to bacterial challenges that is mainly mounted in the salivary glands. Indeed, candidates with a salivary gland-specific expression profile showed a higher response to bacterial challenge at 24 h when compared to sterile PBS-fed mosquitoes, especially when challenged with Gram-positive *S. aureus* bacteria (Figure 5b).

Given the overall subtle variations in the transcriptional abundance of the candidate genes observed by the RTqPCR experiments, we also compared the three replicates to evaluate the general transcript modulation trend, and we observed a constant and reproducible increase of transcript abundance between 5 and 24 h after infectious meals, as shown in Figure S4 and in Figure 3. 

#### 2.3.2. Injection Assays Reveal a Late Transcriptional Increase of SG+ Candidates

The transcriptional modulation of selected salivary genes was further investigated by injecting mosquitoes with sub-lethal doses of bacteria. Again, the time points at 5 and 24 h post challenge, which are indicative of an early and a late response, respectively, were selected to evaluate the modulation of salivary gene expression in the context of a systemic response to invading pathogens.

**Figure 6:**
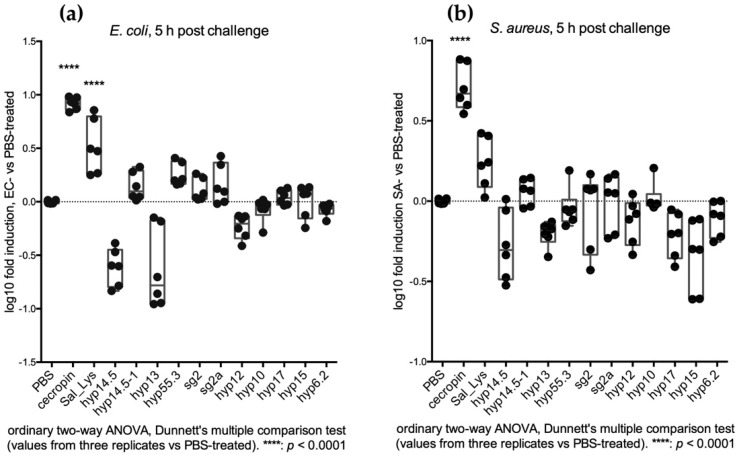
Injection assay: (**a**) transcriptional variations 5 h after infectious EC thoracic injections; (**b**) transcriptional variations 5 h after SA infectious thoracic injections. Biological and technical replicates are reported as dots corresponding to log10 of fold induction (ratio between bacteria-treated and PBS-treated mosquitoes). Statistical analysis (2-way ANOVA followed by Dunnett’s multiple comparison test) was applied on transcript fold change values (ratio between bacteria challenged values and sterile PBS challenged values) and is reported below each graph.

As expected, cecropin expression levels were significantly upregulated soon after bacteria injections [24,38], while the salivary genes did not show relevant variation in abundance soon after the systemic challenge (Figure 6). Noticeably, no increase in the hyp15, hyp17, and hyp6.2 transcript abundance was observed at 5 h after systemic challenge (Figure 6), a result that is different from our previous observations after an SA-infectious meal (Figure 4).

**Figure 7:**
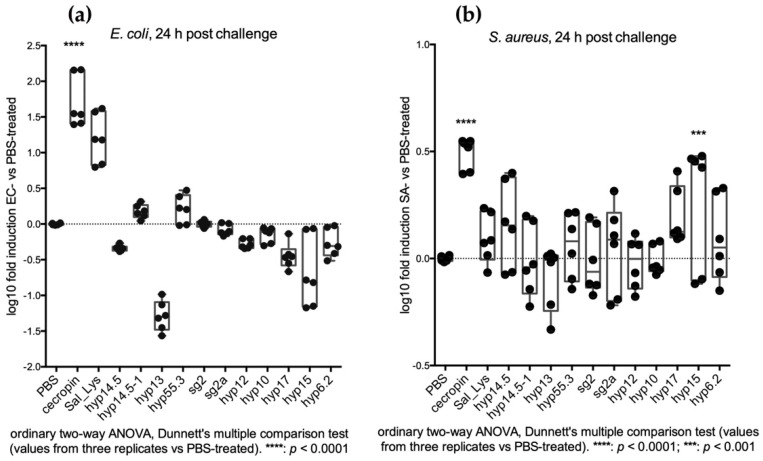
Injection assay: (**a**) transcriptional variations 24 h after infectious EC thoracic injections; (**b**) transcriptional variations 24 h after SA infectious thoracic injections. Biological and technical replicates are reported as dots corresponding to log10 of fold induction (ratio between bacteria-treated and PBS-treated mosquitoes). Statistical analysis (2-way ANOVA followed by Dunnett’s multiple comparison test) was applied on transcript fold change values (ratio between bacteria challenged values and sterile PBS challenged values) and is reported below each graph.

In the first 24 h after the systemic challenge by bacterial thoracic injections, we could observe a further increase in the expression of ubiquitous AMPs (such as cecropin) and a significant upregulation of salivary hyp15 exclusively upon SA challenge (Figure 7).

Overall, we could not reveal any increase in gene transcription at 24 h after the injection of Gram-negative bacteria. On the contrary, most of our candidate genes resulted as being upregulated after the challenge with the Gram-positive *S. aureus*; in particular, hyp15 and hyp14.5 showed a strong and significant increase in transcript abundance in the first 24 h after the challenge (Figure 7 and Figure S5).

## 3. Discussion

Mosquito salivary glands play a tripartite role in mosquito physiology, representing a key organ in nutrient acquisition, pathogen transmission, and immunity. Mosquito saliva is secreted during meal acquisition and is of crucial importance in the feeding processes by virtue of the rich mixture of molecules that it contains. Female mosquito saliva contains molecules that are primarily aimed to counteract the hemostatic response of the vertebrate host, while the saliva of both male and female mosquitoes contains factors carrying digestive properties. Part of the saliva content is ingested by the mosquito during feeding and can exert its biochemical activity in the crop and/or in the midgut. In this context, the local response of innate mosquito immunity to invading pathogens during sugar and/or blood feeding has been poorly understood so far: indeed, limited data are available regarding the transcriptional regulation of known immune factors upon an infectious meal compared to systemic infections [26]. It has been previously shown that the expression of known AMPs (such as, for instance, defensins and cecropins) are not activated immediately after an infectious sugar meal in the organs analysed (such as midgut, salivary glands, hemocytes, and fat body cells). Instead, they often show a temporary decrease in abundance shortly after challenge [26]. Since the salivary factors directly involved in mosquito innate immunity have not been described so far and considering that a relevant number of salivary genes (around 40%) do not show any sequence similarity with known proteins, it has been proposed that some of these salivary proteins/peptides may have antimicrobial properties [1,3]. 

In this study we investigated the transcriptional modulation of selected *An. coluzzii* salivary gland genes upon both local and systemic immune stimulations. The experimental pipeline of this work (Figure 1) originates from several observations indicating that expression of immunity-related genes may be modulated by immune challenges [39]. Most of the immune response of mosquitoes to infection by different pathogens rely on the activation of specific immune pathways and is accomplished by immune cellular component of the hemolymph, hemocytes, and fat body cells [40]. The specific role of local immunity in the defence against invading pathogens has been more recently investigated in mosquitoes [26], highlighting the active role of the midgut in the immune defence and the interorgan communications between immune cells (i.e., hemocytes and fat body cells) and the other mosquito tissues, such as midgut and salivary glands. Here, we selected a short list of 11 salivary candidates and analysed their transcriptional modulation upon local (infectious meal) and systemic (intrathoracic microinjections) immune challenges, thus providing indications of possible activity in the defence against bacteria. 

Interestingly, the transcript abundance of the three SG+ candidate genes (*hyp17*, *hyp15* and *hyp6.2*) appeared to increase early (5 h) after SA infection by sugar feeding, which suggests their possible involvement in an initial local immune response to Gram-positive bacterial challenge (Figure 4); notably, this increase was statistically significant for hyp15 (Figure 4b,d). Indeed, as reported in the literature, SA (Gram-positive bacteria) elicits a general higher immune response. While the salivary-enriched *hyp17*, *hyp15*, and *hyp6.2* show the highest response to the infectious meal challenge (especially with SA) at the early time point (5 hpc), their response to the systemic challenge (SA) appeared to shift at the later time point (24 hpc). This observation may be interpreted as the result of immune crosstalk between tissues, supporting a role of the salivary glands as an immune organ participating in the protective systemic response against bacteria growing in the hemocoel at the tissue level. Moreover, our observations indicate that the abundance of the salivary genes selected in our study was modulated by bacterial infection, especially upon EC stimulus, with a general increase observed between 5 and 24 h after the infectious meal when compared to sterile PBS-fed mosquitoes.

We believe that these data may help characterize novel antimicrobial activities and point out that the triggering of innate immune defence mechanisms may happen with at different times when they are induced by means of local challenge (infectious meal) or systemic challenge (thoracic injection) and depending on the specific pathogen (i.e., Gram-negative vs. Gram-positive bacteria). However, it should be also taken into consideration that the alteration of salivary candidate expression may be part of a more general metabolic response to the infectious meal. In conclusion, two genes (*hyp14.5* and *hyp15*) were induced by bacteria injection, while the modulation of three genes (*hyp15*, *hyp12* and *sg2*) resulted from bacteria feeding. *Sg2* and *sg2a*, which are part of the same small gene cluster and likely originated through gene duplications, are glycine-rich (17.5%–24.3%), a molecular feature that is typically found in antimicrobial peptides from different insect species, for example, the gloverins from the silkworm *Bombyx mori* (Figure S3) [41,42,43]. On the other hand, the acidic candidates *hyp12* and *hyp10*, which also probably originate from a gene duplication, share four conserved cysteines and a similar secondary structure with two predicted alpha-helices (Figure S2). Finally, the basic salivary gland-enriched *hyp15* and *hyp17*, which are also glycine-rich (16.7–20.8%, respectively), show a predicted random coil structure with a short predominantly alpha-helix single region (Figure S1) that is also found in different classes of antimicrobial peptides [41].

Overall, our data suggest that the mosquito immune system can discern between local and systemic stimuli. Indeed, local and systemic microbial infections seem to affect the expression of salivary candidates differently, highlighting that their transcriptional activation depends on the nature of immune stimulation. This work investigates, for the first time, gene transcriptional modulation at the SG level upon immune challenges. Moreover, we reported one of the first pieces of molecular evidence of the connection between different immune stimulations and the regulation of expression in the salivary glands of both tissue-specific genes and genes with a more ubiquitous expression. Notably, we only found a few genes whose expression was significantly modulated by immune challenge; however, the absence of the transcriptional modulation of other salivary candidates does not exclude their possible role in the defence against bacteria on its own. Further functional analyses will certainly be needed to elucidate the effective involvement of these salivary candidates in immune activity, and we believe that the work reported here provides useful indications toward this direction.

## 4. Materials and Methods

### 4.1. Bioinformatic Analysis and Candidate Selection

From the salivary transcriptome [16,29], we selected a list of 11 putative AMPs based on expression profiles and sequence properties. In detail, we selected genes whose expression was (i) specific or enriched in female salivary glands, (ii) restricted to both male and female salivary glands, or (iii) abundant in salivary glands of both sexes but also extended to other tissues. A second selection parameter was based on predicted amino acid length and composition (percentage of Gly, Cys, Pro), the acidic or basic nature of the peptide, predicted hydrophobicity, and secondary structure. Signal peptides were predicted using the SignalP prediction tool (http://www.cbs.dtu.dk/services/SignalP/) (accessed on 11 June 2021); molecular weights and isoelectric points were calculated at http://web.expasy.org/compute_pi/ (accessed on 11 September 2021); secondary structure predictions were obtained by PSIPRED (http://bioinf.cs.ucl.ac.uk/psipred/) (accessed on 11 June 2021); secondary proteolytic cleavage sites (pro-peptides) were predicted using the ProP 1.0 and Spider P tools at http://www.cbs.dtu.dk/services/ProP/ (accessed on 1 July 2019) and http://www.arachnoserver.org/spiderP.html (accessed on 1 July 2019), respectively. Finally, potential antimicrobial activities were assessed using the CAMPR3-RF prediction tool (http://www.camp.bicnirrh.res.in) (accessed on 10 June 2021).

### 4.2. Mosquito Rearing

*An. coluzzii* mosquitoes (GA-CAM strain, *An. gambiae* “M” form, originally collected in Cameroon) were reared in the insectary of the Department of Public Health and Infectious Diseases at Sapienza University under standard conditions (28 °C, 80% relative humidity, 12:12 h light:dark photoperiod). Mosquito cycle maintenance was achieved by sugar feeding on cotton swabs with a 10% sucrose solution placed at the top of the cage and by means of membrane feeding using defibrinated mutton blood. Adult female mosquitoes 3–5 days post-emergence (dpe), not jet blood fed, were used for all of the experiments (local and systemic immune challenges). In the case of the local immune challenge, the mosquitoes were starved overnight before the infectious sugar meal was provided.

### 4.3. Immune Challenges

Transcriptional modulation of the candidate genes was investigated by RTqPCR by challenging mosquitoes with Gram-negative (*Escherichia coli*) or Gram-positive (*Staphylococcus aureus*) bacteria. Systemic infection was induced by bacterial microinjection in the mosquito thorax, while local infection was stimulated by infected sugar meals. 

Bacterial growth: *E. coli* (EC, ATCC 25922) and *S. aureus* (SA, ATCC 25923) were initially streaked on LB agar plates from 20% glycerol stocks and were incubated overnight at 37 °C. Single colonies from *E. coli* and *S. aureus* agar plates were inoculated in 20 mL LB medium and were grown overnight in a shaking incubator at 250 rpm and 37 °C. Bacterial density was determined measuring the absorbance of serial dilutions at 600 nm. Bacterial dilutions with an OD_600_ of 0.5/mL and 0.2/mL were selected for local and systemic stimulations, respectively.

Systemic infection (thoracic injection): Aliquots of *E. coli* and *S. aureus* cultures (700 μL 0.2 OD_600_/mL) were centrifuged at 500 g, washed with sterile phosphate-buffered saline (PBS, 0.2 μm filtered), and resuspended in 700 μL of PBS. Adult female mosquitoes (3–5 dpe) were briefly anesthetized on ice and 50 nL of a bacterial suspension of *E. coli* or *S. aureus* (0.2 OD/mL in PBS) were injected into the mosquito thorax through a Drummond^TM^ microinjector (Nanoject II, equipped with 3.5” glass capillaries Drummond, 3000 203 G/X). A group of control mosquitoes was injected with 50 nL of sterile PBS. A total of 30–40 female mosquitoes were injected with PBS, *E. coli* (EC), or *S. aureus* (SA). Mosquitoes were then allowed recover in standard insectary conditions, and six mosquitoes for treatment were collected at 5- and 24-h post-injection and were stored at −80 °C. Three independent experimental biological replicates were performed. 

Local infection (blue methylene sugar feeding): Aliquots of *E. coli* and *S. aureus* bacterial culture (500 µL, 0.5 OD/mL) were washed in sterile PBS as above and were resuspended in 10% sucrose plus 0.5% methylene blue in PBS. Female mosquitoes 3–4 days post-emergence were starved overnight and were then allowed to feed on cotton swabs imbibed with bacterial infected sucrose and methylene blue for two hours. A group of control mosquitoes was fed on sterile 10% sucrose and 0.5% methylene blue solution in PBS. A total of 40–50 female mosquitoes was used for each group. Fed mosquitoes (recognized by the blue abdomen under a stereoscope) were selected (Figure 2). From these subgroups (around 25–30 fed mosquitoes/group), five mosquitoes for each treatment (PBS, EC, SA) were collected at 5 h and 24 h after the infected sugar meal and were stored at −80 °C. As for the injection experiment, three independent experimental replicates were performed. 

### 4.4. Transcriptional Analysis

Total RNA was extracted from the pools of whole females (six or five mosquitoes for each TP/condition) using Trizol* Reagent (Life Technologies, Carlsbad, CA, USA) according to the manufacturer’s protocols. RNA quantity and quality were assessed by both spectrophotometry (BioTek SynergyHT, Take 3) and gel electrophoresis. For each sample, 10 μg of total RNA were treated with TURBO^TM^ DNase (Ambion TURBO DNA-*free*^TM^ kit) to remove genomic DNA contamination. The treatment efficacy was verified through standard PCR designed to amplify the *An. coluzzii* rpS7 transcript (AGAP010592), which encodes the 40S ribosomal protein S7. Aliquots of DNase-treated total RNA (2 μg) were used for first-strand cDNA synthesis using the SuperScript^TM^ II Reverse Transcriptase (Invitrogen) and Oligo(dT)_18-20_ primers. The cDNA samples (100 ng/μL) were diluted to 10 ng/μL and were used as a template for quantitative real-time PCR. The relative quantification procedure was applied and included a standard curve for each target gene and for the endogenous reference gene (rpS7) in the amplification reactions. In detail, the cDNA samples used for the preparation of the standard curves were obtained from entire naïve female mosquitoes following the procedure described before. Serial 1:5 cDNA dilutions were prepared to obtain 5 points for each standard curve at the respective concentrations: (100 ng/μL), (20 ng/μL), (4 ng/μL), (0.8 ng/μL), and (0.16 ng/μL). Each qPCR reaction consisted of the following mixture (20 μL total volume): 2 μL of cDNA (C_f_ = (1 ng/μL)), 10 μL of 2X PowerUp^TM^ SYBR^®^ Green Master Mix, and 4 μL of forward and reverse primers (C_i_ = (1 μΜ), C_f_ = (200 nM)). The PCR thermal cycle included an initial holding stage of 10 min at 95 °C followed by 40 amplification cycles (15 s at 95 °C, 1 min at 60 °C). A melt curve stage analysis was introduced to assess primer pair efficiency and to confirm the production of the specific amplicon for each target gene. Each of the three independent biological replicates was analyzed in duplicate on the same amplification reaction. The relative quantification analysis was conducted by interpolating the C_T_ value of each candidate with the standard curve and then by normalizing it with the relative value of the rpS7 endogenous reference. Data from the mosquitoes at 5 and 24 h after an uninfected sugar meal or sterile PBS injection (internal control samples) were placed as a calibrator sample. The primers used for the PCR amplifications are reported below (Table 3). Graphs and statistical analyses were performed using GraphPad Prism software; box and whisker plots contain these data: the first quartile, Q1, is at the far bottom of the box; the median is shown as a line inside the box; the third quartile, Q3, is shown near the far top of the box; minimum and maximum values are also indicated. Statistical analyses are reported in the legend of the Figures.

## Figures and Tables

**Table 1:** List of putative antimicrobial salivary candidates. Gene names and AGAP codes are reported in the first two columns. Expression profile tab indicates genes with expression that is (i) enriched in the female glands (SG+), (ii) restricted to female and male glands (SG F/M), and (iii) ubiquitous (U). The length of the predicted signal peptide (SP), the length (AA), the molecular weight (kDa), and the isoelectric point (pI) of the predicted peptide (full, precursor, or mature forms) are reported.

Genes		Expression Profile			Full		Precursor				Mature		
Name ^1^	AGAP ID	SG+	SG F/M	U	AA	kDa	SP	AA	kDa	pI	AA	kDa	pI
hyp6.2 secreted salivary basic pep.	AGAP006495	X			85	9.3	27	58	6.3	10.41	35	4.0	11.57
hyp17 hypothetical salivary pr. 17	AGAP000151	X			77	8.1	29	48	4.9	10.83			
hyp15 hypothetical salivary pr. 15	AGAP000152	X			78	9.9	30	48	4.9	10.55			
sg2a salivary protein	AGAP006504		X		173	17.6	18	155	15.6	7.02	54	5.2	5.52
sg2 salivary protein	AGAP006506		X		114	11.8	20	94	9.7	3.49			
hyp55.3 putative 55.3 salivary pr.	AGAP005822		X		513	55.2	21	492	52.9	8.73	276	29.8	8.77
hyp12 hypothetical salivary pr. 12	AGAP008306		X		92	10.0	21	71	7.9	4.47			
hyp10 hypothetical salivary pr. 10	AGAP008307		X		90	10.0	19	67	7.5	5.42	63	7.0	5.77
hyp14.5 sim. to Cx. 14.5 sal. Pep.	AGAP004883			X	180	19.7	26	154	16.8	8.07			
hyp14.5-1 sim. to Cx. 14.5 sal. Pep.	AGAP001174			X	154	17.2	20	134	14.9	6.15			
hyp13 hypothetical pr. 13	AGAP003474			X	56	6.2	22	34	3.8	4.75	19	2.0	3.77
Ag_sal_Lyzo1 sal. Lysozyme	AGAP007347			X	140	15.3	20	120	13.3	8.91			
Cecropin	AGAP000693			X	58	6.1	23	35	3.6	10.79			

^1^ Abbreviations: pep.: peptide; pr.: protein; sal.: salivary; sim.: similar; Cx.: Culex.

**Table 2:** Prediction of antimicrobial activity as calculated by the CAMPR3-RF online tool.

CAMP R3—Prediction of Antimicrobial Peptides								
SVM ^1^		RF ^1^		DA ^1^		ANN ^1^		
PRE	MAT	PRE	MAT	PRE	MAT	PRE	MAT	
0.057	0.193	0.062	0.304	0.006	0.048	NAMP	NAMP	**hyp6.2_AGAP006495**
0.062	0.062	0.055	0.055	0.015	0.015	NAMP	NAMP	hyp17_AGAP000151
0.092	0.092	0.149	0.149	0.028	0.028	AMP	AMP	hyp15_AGAP000152
0.316	0.316	0.022	0.022	0.006	0.006	NAMP	NAMP	hyp12_AGAP008306
0.298	0.269	0.099	0.052	0.088	0.166	AMP	AMP	**hyp10_AGAP008307**
1.000	0.445	0.917	0.504	1.000	0.102	NAMP	NAMP	**sg2a_AGAP006504**
0.324	0.324	0.858	0.858	0.937	0.937	NAMP	NAMP	sg2_AGAP006506
1.000	1.000	0.913	0.932	0.000	1.000	AMP	AMP	**hyp55.3_AGAP005822**
1.000	1.000	0.945	0.945	1.000	1.000	AMP	AMP	hyp14.5_AGAP004883
1.000	1.000	0.970	0.970	1.000	1.000	AMP	AMP	hyp14.5-1_AGAP001174
0.601	0.579	0.287	0.115	0.080	0.189	NAMP	NAMP	**hyp13_AGAP003474**
1.000	1.000	0.873	0.873	1.000	1.000	AMP	AMP	LysC1_AGAP007347
0.985	0.985	0.997	0.997	0.999	0.999	AMP	AMP	Cec_AGAP000693

^1^ Results obtained with four different algorithms (SVM, support vector machine; RF, random forest classifier; DA, discriminant analysis; ANN, artificial neural network) are reported. Numbers represent the probability (1, highest probability, dark red; 0, lowest probability, light red) of exerting antimicrobial activity; in the last two columns, only the prediction of whether antimicrobial activity (AMP) is exerted or not (NAMP) is reported. Genes with a predicted maturation proteolytic cleavage are in bold (PRE, precursor; MAT, mature form). Both graphical styles were chosen to provide information: Bold indicates a specific subset of genes (as indicated in the Table legend); red gradient corresponds to statistical probability (as indicated in the Table legend).

**Table 3:** List of primers used in this work. Gene names and AGAP codes (Vector base, VB ID) are reported in the first two columns. The forward (pF) and reverse primer (pR) names with their respective oligonucleotide sequences (qPCR F and qPCR R) are listed. In the last column, the amplicon size for each primer pair is reported.

GENE ^1^	VB ID	pF	qPCR F	pR	qPCR R	SIZE
hyp6.2 secreted sal. basic pep.	AGAP006495	06495_F	CATTGCTTGTGGTGCTGTCC	06495_R	AAGTGCTGCCGACATTACCA	87
hyp17 hypothetical sal. pr. 17	AGAP000151	00151_F	TGTCTGCTGCTCTTCATCGC	00151_R	TGGGGTCGCTCTTTTGTCAT	99
hyp15 hypothetical sal. pr. 15	AGAP000152	00152_F	GGGCAGAGACCGAAATACCA	00152_R	CCAGTCAGTGCCATCCTAGC	99
sg2a salivary protein	AGAP006504	06504_F	AAATGGTCAGCAAGGACGAG	06504_R	ATGCCTCCATTCTGTTGTCC	96
sg2 salivary protein	AGAP006506	06506_F	TGCCGAACCTTGGTAATCTG	06506_R	ACGCATCGGTAAAGTTCGTC	96
hyp55.3 putative 55.3 sal. pr.	AGAP005822	05822_F	GACGGCAAGAAAGTTGAAGC	05822_R	TGACGTTGGTGAGTCGTTTG	104
hyp12 hypothetical sal. pr. 12	AGAP008306	08306_F	AGAAACTGCAGCTCACGAAC	08306_R	TGCACAGAGCACAACAACAG	78
hyp10 hypothetical sal. pr. 10	AGAP008307	08307_F	GTCACCATGGAAGACCCCCGTACCGAGCT	08307_R	GTCACCCGGGGCGAATATCCTTTGTACAGT	204
hyp14.5 sim. to Cx 14.5 sal. pep.	AGAP004883	04883_F	GCTCAAAAAGCTTCGCAGAG	04883_R	TCGGATTATCTGGCAGGAAG	102
hyp14.5-1 sim. to Cx 14.5 sal. pep.	AGAP001174	01174_F	TCTACAAGGCGCAGAATGTG	01174_R	TGGGCCGAATTACACTCATC	100
hyp13 hypothetical pr. 13	AGAP003474	03474_F	GTCACCATGGGGAACGAAATCATACAAAA	03474_R	GTCACCCGGGTTGCGATCCGGAGTCACTGT	105
Ag_sal_Lyzo1 sal. lysozyme	AGAP007347	07347_F	ACGGCATCTTCCAGATCAAC	07347_R	GACGTTTGTGGATCAGCTTG	138
Cecropin	AGAP000693	00693_F	GTCACCATGGGACGGCTGAAGAAGCTGGG	00693_R	GTCACCCGGGACCGAGCGCCTTAACGCCTG	104
Ag_RpS7	AGAP010592	AgS7_qF	GTGCGCGAGTTGGAGAAGA	AgS7_qR	ATCGGTTTGGGCAGAATGC	77

^1^ Abbreviations: pep.: peptide; pr.: protein; sal.: salivary; sim.: similar; Cx.: Culex.

## Data Availability

All the data generated during this work are reported in the manuscript. Aminoacid sequences are available at the VectorBase web site: https://vectorbase.org/vectorbase/app (Accessed on 10 May 2021).

## Supplementary Materials

The following are available online at www.mdpi.com/article/10.3390/pathogens10101300/s1, Figure S1: Secondary structure predicted for SG+ candidates hyp6.2, hyp15, and hyp17; Figure S2: hyp10 and hyp12; Figure S3: Sg2/sg2a/AGAP006505 gene cluster; Figure S4: Feeding assay: comparison of replicates; Figure S5: Injection assay: candidate transcriptional modulation between 5 and 24 h post challenge; Dataset S1: FASTA sequences.

## References

[1] Ribeiro J., Arcà B. (2009). From Sialomes to the Sialoverse: An Insight into Salivary Potion of Blood-Feeding Insects. Adv. Insect Phys..

[2] Stoffolano J.G., Haselton A.T. (2013). The Adult Dipteran Crop: A Unique and Overlooked Organ. Annu. Rev. Entomol..

[3] Ribeiro J.M.C., Mans B.J., Arca B. (2010). An insight into the sialome of blood-feeding Nematocera. Insect Biochem. Mol. Biol. Engl..

[4] Ribeiro J.M.C., Martin-Martin I., Arcà B., Calvo E. (2016). A Deep Insight into the Sialome of Male and Female *Aedes aegypti* Mosquitoes. PLoS ONE.

[5] Moreira-Ferro C.K., Daffre S., James A., Marinotti O. (1998). A lysozyme in the salivary glands of the malaria vector *Anopheles darlingi*. Insect Mol. Biol..

[6] Dimopoulos G., Seeley D., Wolf A., Kafatos F.C. (1998). Malaria infection of the mosquito *Anopheles gambiae* activates immune-responsive genes during critical transition stages of the parasite life cycle. EMBO J..

[7] James A., Rossignol P. (1991). Mosquito salivary glands: Parasitological and molecular aspects. Parasitol. Today.

[8] Foster W.A. (1995). Mosquito Sugar Feeding and Reproductive Energetics. Annu. Rev. Entomol. Annu. Rev..

[9] Stoffolano J.G., Jurenka R. (2019). Chapter Two—Fly Foregut and Transmission of Microbes.

[10] Rosinski-Chupin I., Briolay J., Brouilly P., Perrot S., Gomez S.M., Chertemps T., Roth C.W., Keime C., Gandrillon O., Couble P. (2006). SAGE analysis of mosquito salivary gland transcriptomes during Plasmodium invasion. Cell. Microbiol..

[11] Chowdhury A., Modahl C.M., Tan S.T., Xiang B.W.W., Missé D., Vial T., Kini R.M., Pompon J.F. (2020). JNK pathway restricts DENV2, ZIKV and CHIKV infection by activating complement and apoptosis in mosquito salivary glands. PLoS Pathog..

[12] Pinheiro-Silva R., Borges L., Coelho L.P., Cabezas-Cruz A., Valdés J.J., Rosário V.D., De La Fuente J., Domingos A. (2015). Gene expression changes in the salivary glands of *Anopheles coluzzii* elicited by *Plasmodium berghei* infection. Paras. Vect..

[13] Ribeiro J.M., Francischetti I.M. (2003). Role of arthropod saliva in blood feeding: Sialome and post-sialome perspectives. Annu. Rev. Entomol..

[14] Lanfrancotti A., Lombardo F., Santolamazza F., Veneri M., Castrignanò T., Coluzzi M., Arcà B. (2002). Novel cDNAs encoding salivary proteins from the malaria vector *Anopheles gambiae*. FEBS Lett..

[15] Arcà B., Lombardo F., Capurro M., della Torre A., Dimopoulos G., James A., Coluzzi M. (1999). Trapping cDNAs encoding secreted proteins from the salivary glands of the malaria vector *Anopheles gambiae*. Proc. Natl. Acad. Sci. USA.

[16] Arcà B., Lombardo F., Struchiner C.J., Ribeiro J.M.C. (2017). Anopheline salivary protein genes and gene families: An evolutionary overview after the whole genome sequence of sixteen Anopheles species. BMC Genom..

[17] Kumar A., Srivastava P., Sirisena P., Dubey S.K., Kumar R., Shrinet J., Sunil S. (2018). Mosquito Innate Immunity. Insects.

[18] Phillips D.R., Clark K.D. (2017). *Bombyx mori* and *Aedes aegypti* form multifunctional immune Complexes that integrate Pattern recognition, melanization, coagulants, and hemocyte recruitment. PLoS ONE.

[19] Bulet P., Hetru C., Dimarcq J.-L., Hoffmann D. (1999). Antimicrobial peptides in insects; structure and function. Dev. Comp. Immunol..

[20] Wu Q., Patočka J., Kuča K. (2018). Insect Antimicrobial Peptides, a Mini Review. Toxins.

[21] Bartholomay L.C., Michel K. (2018). Mosquito Immunobiology: The Intersection of Vector Health and Vector Competence. Annu. Rev. Entomol..

[22] Meister M., Lemaitre B., Hoffmann J.A. (1997). Antimicrobial peptide defense in *Drosophila*. BioEssays.

[23] Lemaitre B., Hoffmann J. (2007). The Host Defense of *Drosophila melanogaster*. Annu. Rev. Immunol..

[24] Christophides G., Zdobnov E., Barillas-Mury C., Birney E., Blandin S., Blass C., Brey P.T., Collins F.H., Danielli A., Dimopoulos G. (2002). Immunity-Related Genes and Gene Families in *Anopheles gambiae*. Science.

[25] Meister S., Kanzok S.M., Zheng X.-L., Luna C., Li T.-R., Hoa N.T., Clayton J.R., White K.P., Kafatos F.C., Christophides G.K. (2005). Immune signaling pathways regulating bacterial and malaria parasite infection of the mosquito *Anopheles gambiae*. Proc. Natl. Acad. Sci. USA.

[26] De T.D., Sharma P., Thomas T., Singla D., Tevatiya S., Kumari S., Schauhan C., Rani J., Srivastava K., Kaur R. (2018). Interorgan molecular communication strategies of “Local” and “Systemic” innate immune responses in mosquito *Anopheles stephensi*. Front. Immunol..

[27] Ramirez J.L., Souza-Neto J., Cosme R.T., Rovira J., Ortiz A., Pascale J.M., Dimopoulos G. (2012). Reciprocal Tripartite Interactions between the *Aedes aegypti* Midgut Microbiota, Innate Immune System and Dengue Virus Influences Vector Competence. PLoS Negl. Trop. Dis..

[28] Hillyer J.F. (2010). Mosquito immunity. Adv. Exp. Med. Biol..

[29] Arcà B., Lombardo F., Valenzuela J.G., Francischetti I.M.B., Marinotti O., Coluzzi M., Ribeiro J.M.C. (2005). An updated catalogue of salivary gland transcripts in the adult female mosquito, *Anopheles gambiae*. J. Exp. Biol..

[30] Mendes-Sousa A.F., Queiroz D.C., Vale V.F., Ribeiro J.M.C., Valenzuela J.G., Gontijo N.F., Andersen J.F. (2016). An Inhibitor of the Alternative Pathway of Complement in Saliva of New World Anopheline Mosquitoes. J. Immunol..

[31] Baker D.A., Nolan T., Fischer B., Pinder A., Crisanti A., Russell S. (2011). A comprehensive gene expression atlas of sex- and tissue-specificity in the malaria vector, *Anopheles gambiae*. BMC Genom..

[32] Vlachou D., Schlegelmilch T., Christophides G., Kafatos F.C. (2005). Functional Genomic Analysis of Midgut Epithelial Responses in Anopheles during Plasmodium Invasion. Curr. Biol..

[33] Gabere M.N., Noble W.S. (2017). Empirical comparison of web-based antimicrobial peptide prediction tools. Bioinformatics.

[34] Waghu F.H., Barai R.S., Gurung P., Idicula-Thomas S. (2015). CAMPR3: A database on sequences, structures and signatures of antimicrobial peptides. Nucl. Acids Res..

[35] Tzou P., Ohresser S., Ferrandon D., Capovilla M., Reichhart J.-M., Lemaitre B., Hoffmann J.A., Imler J.-L. (2000). Tissue-Specific Inducible Expression of Antimicrobial Peptide Genes in Drosophila Surface Epithelia. Immunity.

[36] Agaisse H., Petersen U.-M., Boutros M., Mathey-Prevot B., Perrimon N. (2003). Signaling Role of Hemocytes in *Drosophila* JAK/STAT-Dependent Response to Septic Injury. Dev. Cell.

[37] Hillyer J.F. (2009). Transcription in mosquito hemocytes in response to pathogen exposure. J. Biol..

[38] Vizioli J., Bulet P., Charlet M., Lowenberger C., Blass C., Müller H.-M., Dimopoulos G., Hoffmann J., Kafatos F., Richman A. (2000). Cloning and analysis of a cecropin gene from the malaria vector mosquito, *Anopheles gambiae*. Insect Mol. Biol..

[39] Dimopoulos G., Christophides G., Meister S., Schultz J., White K.P., Barillas-Mury C., Kafatos F.C. (2002). Genome expression analysis of *Anopheles gambiae*: Responses to injury, bacterial challenge, and malaria infection. Proc. Natl. Acad. Sci. USA.

[40] Baxter R.H.G., Contet A., Krueger K. (2017). Arthropod Innate Immune Systems and Vector-Borne Diseases. Biochemistry.

[41] Yi H.-Y., Chowdhury M., Huang Y.-D., Yu X.-Q. (2014). Insect antimicrobial peptides and their applications. Appl. Microbiol. Biotechnol..

[42] Yi H.-Y., Deng X.-J., Yang W.-Y., Zhou C.-Z., Cao Y., Yu X.-Q. (2013). Gloverins of the silkworm *Bombyx mori*: Structural and binding properties and activities. Insect Biochem. Mol. Biol..

[43] Tian C., Gao B., Fang Q., Ye G., Zhu S. (2010). Antimicrobial peptide-like genes in *Nasonia vitripennis*: A genomic perspective. BMC Genom..

