# Supplementary material for: Effects of Local and Systemic Immune Challenges on the Expression of Selected Salivary Genes in the Malaria Mosquito Anopheles coluzzii
Source: Pathogens. 2021 Oct 9;10(10):1300. doi: 10.3390/pathogens10101300 (PMC8540153; doi:10.3390/pathogens10101300)
Supplement: Supplementary file 1 [file pathogens-10-01300-s001.zip › Dataset_S1.pdf]

Dataset S1: FASTA sequences

FULL LENGTH FASTA SEQUENCES

aminoacid sequences downloaded from VectorBase: https://vectorbase.org/vectorbase/app

In yellow: predicted Signal Peptides according to SignalP Server:

http://www.cbs.dtu.dk/services/SignalP/

In light blue: predicted pro-peptides according to ProP 1.0 and Spider P (mature peptides are listed in red):

http://www.cbs.dtu.dk/services/ProP/,

http://www.arachnoserver.org/spiderP.html

```
>hyp6.2_AGAP006495
MGRVMCLLRLMSTLLVVLSIVGKKTNAAPQVTEAPGNVGSTYSPMADIGRLATGATKLFGQFWNTGTRFGTELSRRTFDF
LRVKK
>hyp17_AGAP000151
MKLYSGYRLLVLLVMTVCCLLLFIAPTGADPLPGQTQRTLGYRGNDKRATPPMHSLGSGARMAMTGGGILGGIFSAL
>hyp15_AGAP000152
MKFYSVGKLVKVLLVMAVCCLLLCTAPTGADPLPGRDRNTIANKSKDKKASAPKHSLGTGARMALTGGGVLGGVLTNM
>hyp12_AGAP008306
MRFCCVALIGLLLCSVQSVTANDPVDALGACSGNLFGLLMTRLQQMVEDFTACRQEATANDPQHDCSDSIQRAKVDLQQQ
LVNYSYCTKNIQ
>hyp10_AGAP008307
MRFLTVLTVGLLVWVGVFATVNAEDPRTELIGCGSVLFHLAANRLSLQLEEFAVCKRSNPGYDCSDSIHRAISDLQQGLF
DLNHCTKDIR
>sg2a_ AGAP006504
MKLFLTLLLTLSVVMVFALPAHHHSRGGDGSSANSTGQSYNNSGSPNNSAGVPDFGFNSQSNVPGFGNGQQPGQQQQGQQ
GQGFPFFGQGQSGFPSFGNRQPFFGQNQQGQDGNGQQGRGIPFFGQGGGQGGIPSFGSGQQNGGIPSLGNGQAQSGFPSF
GNGQQGGNFPFFG
>sg2_ AGAP006506
MKSILVTFATLSVALVVVVAIPTSFNYGGGGGYFINGTGQSFNFSGESNGTSIPGVPDFGSFLPNLGNLTQQFGGSSGAF
PQFSIPSWTNFTDAFSSIFPFFGNVQGGGFPFFG
>hyp55.3_AGAP005822
MKYRFALLTVLVAVVPRPSAALPDFAVPARMTGVASGSLDSSGNVVQNTARVLHLLDQFQSITESLYGLQTPTLYRLATG
LRVVLDSLVESGSPIFQGLSNAARLSTGNITTVFDGIRRSINGTIALHELHQSALNGTVLLLGTGGVHNISTVLHQLVRN
VANLTGALDEIEPAIVAIQEQELSRPTQAQVDGRYPRAAIRRLNGILLDYVNIGQATVPQINAVVNRIRMMDGFIGRLAA
VTGGLWSDLNRTLAAVNGSVYAGVQQRLHTTARTIRNSFNATVLGTARKLKLFFLDDLEELQLVARNASALLVKRLTNVT
GALDGLVNRTEVRLEGYESELVLNRTIAAVTDLAWRLALSVTSSVPGADICYARYNYEFDKIPRLIYGSLVACGQGEART
LQSVGGGLSGALSLTQAQLDAETSNYEQCLGGLVPGSSDVLKRQRIVCLRGAERFSGVLGDAVVDAQLSAFQALLQKELS
YSAERHRQCVRTSGHLMVAEVKRLWSSIANCLS
>hyp14.5_AGAP004883
MLCSRSSTLYAVFAFIFVSVLHDAMALQCRNCLSSKSFAECEKMGEVVVCNSTIVNQNHQAFLPDNPTLPQGNWTEFKCY
QLQVARLHQNNTDSGLRGYARGCTFLHVNFCNGWTSTLNLTSCSTCTSDNCEQHLPTVAPELTTTTTTKKPSSNGANVLT
WRNLSSLGCLSAVVLLLAAR
>hyp14.5-1_AGAP001174
MKLFLLSVFFFLASFQAAQALRCMQGTEIVLPSDDSAEKDKDKVPEFTECSILTTGASAASLLVLKPNTATLPSTDYKCY
HLRIEESESKKATIVKGCIYKAQNVCDGKFKVDTVREVFCSQCDQDECNSAHRFASNWKILSLTLFTILCLFMK
>hyp13_AGAP003474
MKNVFFALLLVVLVCCLVSVQGNEIIQNVVKRSIPLRQLILQHNALDDSDSDSGSQ
>LysC1_AGAP007347
MKVFSTVLLAIVACCAVAEAKTFGKCELAKALANNGIAKASLPDWVCLVQNESAFSTSATNKNKNGSTDYGIFQINNKYW
CDSGYGSNDCKIACKNLLNDDITDDIKCAKLIHKRHGFNAWYGWKNHCNGKKLPNVSSCF
>Cec_AGAP000693
MNFSKIFIFVVLAVLLLCSQTEAGRLKKLGKKIEGAGKRVFKAAEKALPVVAGVKALG
```

PRECURSORS WITHOUT SP

>hyp6.2_AGAP006495
APQVTEAPGNVGSTYSPMADIGRLATGATKLFGQFWNTGTRFGTELSRRTFDFLRVKK
>hyp17_AGAP000151
DPLPGQTQRTLGYRGNDKRATPPMHSLGSGARMAMTGGGILGGIFSAL
>hyp15_AGAP000152
DPLPGRDRNTIANKSKDKKASAPKHSLGTGARMALTGGGVLGGVLTNM
>hyp12_AGAP008306
NDPVDALGACSGNLFGLLMTRLQQMVEDFTACRQEATANDPQHDCSDSIQRAKVDLQQQLVNYSYCTKNIQ
>hyp10_AGAP008307
TVNAEDPRTELIGCGSVLFHLAANRLSLQLEEFAVCKRSNPGYDCSDSIHRAISDLQQGLFDLNHCTKDIR
>sg2a_ AGAP006504
LPAHHHSRGGDGSSANSTGQSYNNSGSPNNSAGVPDFGFNSQSNVPGFGNGQQPGQQQQGQQGQGFPFFGQGQSGFPSFG
NRQPFFGQNQQGQDGNGQQGRGIPFFGQGGGQGGIPSFGSGQQNGGIPSLGNGQAQSGFPSFGNGQQGGNFPFFG
>sg2_ AGAP006506
IPTSFNYGGGGGYFINGTGQSFNFSGESNGTSIPGVPDFGSFLPNLGNLTQQFGGSSGAFPQFSIPSWTNFTDAFSSIFP
FFGNVQGGGFPFFG
>hyp55.3_AGAP005822
LPDFAVPARMTGVASGSLDSSGNVVQNTARVLHLLDQFQSITESLYGLQTPTLYRLATGLRVVLDSLVESGSPIFQGLSN
AARLSTGNITTVFDGIRRSINGTIALHELHQSALNGTVLLLGTGGVHNISTVLHQLVRNVANLTGALDEIEPAIVAIQEQ
ELSRPTQAQVDGRYPRAAIRRLNGILLDYVNIGQATVPQINAVVNRIRMMDGFIGRLAAVTGGLWSDLNRTLAAVNGSVY
AGVQQRLHTTARTIRNSFNATVLGTARKLKLFFLDDLEELQLVARNASALLVKRLTNVTGALDGLVNRTEVRLEGYESEL
VLNRTIAAVTDLAWRLALSVTSSVPGADICYARYNYEFDKIPRLIYGSLVACGQGEARTLQSVGGGLSGALSLTQAQLDA
ETSNYEQCLGGLVPGSSDVLKRQRIVCLRGAERFSGVLGDAVVDAQLSAFQALLQKELSYSAERHRQCVRTSGHLMVAEV
KRLWSSIANCLS
>hyp14.5_AGAP004883
LQCRNCLSSKSFAECEKMGEVVVCNSTIVNQNHQAFLPDNPTLPQGNWTEFKCYQLQVARLHQNNTDSGLRGYARGCTFL
HVNFCNGWTSTLNLTSCSTCTSDNCEQHLPTVAPELTTTTTTKKPSSNGANVLTWRNLSSLGCLSAVVLLLAAR
>hyp14.5-1_AGAP001174
LRCMQGTEIVLPSDDSAEKDKDKVPEFTECSILTTGASAASLLVLKPNTATLPSTDYKCYHLRIEESESKKATIVKGCIY
KAQNVCDGKFKVDTVREVFCSQCDQDECNSAHRFASNWKILSLTLFTILCLFMK
>hyp13_AGAP003474
NEIIQNVVKRSIPLRQLILQHNALDDSDSDSGSQ
>LysC1_AGAP007347
KTFGKCELAKALANNGIAKASLPDWVCLVQNESAFSTSATNKNKNGSTDYGIFQINNKYWCDSGYGSNDCKIACKNLLND
DITDDIKCAKLIHKRHGFNAWYGWKNHCNGKKLPNVSSCF
>Cec_AGAP000693
GRLKKLGKKIEGAGKRVFKAAEKALPVVAGVKALG

MATURE PEPTIDES

```
>hyp6.2_AGAP006495
LATGATKLFGQFWNTGTRFGTELSRRTFDFLRVKK
>hyp17_AGAP000151
DPLPGQTQRTLGYRGNDKRATPPMHSLGSGARMAMTGGGILGGIFSAL
>hyp15_AGAP000152
DPLPGRDRNTIANKSKDKKASAPKHSLGTGARMALTGGGVLGGVLTNM
>hyp12_AGAP008306
NDPVDALGACSGNLFGLLMTRLQQMVEDFTACRQEATANDPQHDCSDSIQRAKVDLQQQLVNYSYCTKNIQ
>hyp10_AGAP008307
TELIGCGSVLFHLAANRLSLQLEEFAVCKRSNPGYDCSDSIHRAISDLQQGLFDLNHCTKDIR
>sg2a_ AGAP006504
GIPFFGQGGGQGGIPSFGSGQQNGGIPSLGNGQAQSGFPSFGNGQQGGNFPFFG
>sg2_ AGAP006506
IPTSFNYGGGGGYFINGTGQSFNFSGESNGTSIPGVPDFGSFLPNLGNLTQQFGGSSGAFPQFSIPSWTNFTDAFSSIFP
FFGNVQGGGFPFFG
>hyp55.3_AGAP005822
LAAVTGGLWSDLNRTLAAVNGSVYAGVQQRLHTTARTIRNSFNATVLGTARKLKLFFLDDLEELQLVARNASALLVKRLT
NVTGALDGLVNRTEVRLEGYESELVLNRTIAAVTDLAWRLALSVTSSVPGADICYARYNYEFDKIPRLIYGSLVACGQGE
ARTLQSVGGGLSGALSLTQAQLDAETSNYEQCLGGLVPGSSDVLKRQRIVCLRGAERFSGVLGDAVVDAQLSAFQALLQK
ELSYSAERHRQCVRTSGHLMVAEVKRLWSSIANCLS
>hyp14.5_AGAP004883
LQCRNCLSSKSFAECEKMGEVVVCNSTIVNQNHQAFLPDNPTLPQGNWTEFKCYQLQVARLHQNNTDSGLRGYARGCTFL
HVNFCNGWTSTLNLTSCSTCTSDNCEQHLPTVAPELTTTTTTKKPSSNGANVLTWRNLSSLGCLSAVVLLLAAR
>hyp14.5-1_AGAP001174
LRCMQGTEIVLPSDDSAEKDKDKVPEFTECSILTTGASAASLLVLKPNTATLPSTDYKCYHLRIEESESKKATIVKGCIY
KAQNVCDGKFKVDTVREVFCSQCDQDECNSAHRFASNWKILSLTLFTILCLFMK
>hyp13_AGAP003474
QLILQHNALDDSDSDSGSQ
>LysC1_AGAP007347
KTFGKCELAKALANNGIAKASLPDWVCLVQNESAFSTSATNKNKNGSTDYGIFQINNKYWCDSGYGSNDCKIACKNLLND
DITDDIKCAKLIHKRHGFNAWYGWKNHCNGKKLPNVSSCF
>Cec_AGAP000693
GRLKKLGKKIEGAGKRVFKAAEKALPVVAGVKALG
```
