# Supplementary material for: Effects of Local and Systemic Immune Challenges on the Expression of Selected Salivary Genes in the Malaria Mosquito Anopheles coluzzii
Source: Pathogens. 2021 Oct 9;10(10):1300. doi: 10.3390/pathogens10101300 (PMC8540153; doi:10.3390/pathogens10101300)
Supplement: Supplementary file 1 [file pathogens-10-01300-s001.zip › Figure_S1.pdf]

Figure S1: secondary structure predicted for SG+ candidates hyp6.2, hyp15 and hyp17.

Hyp.6.2

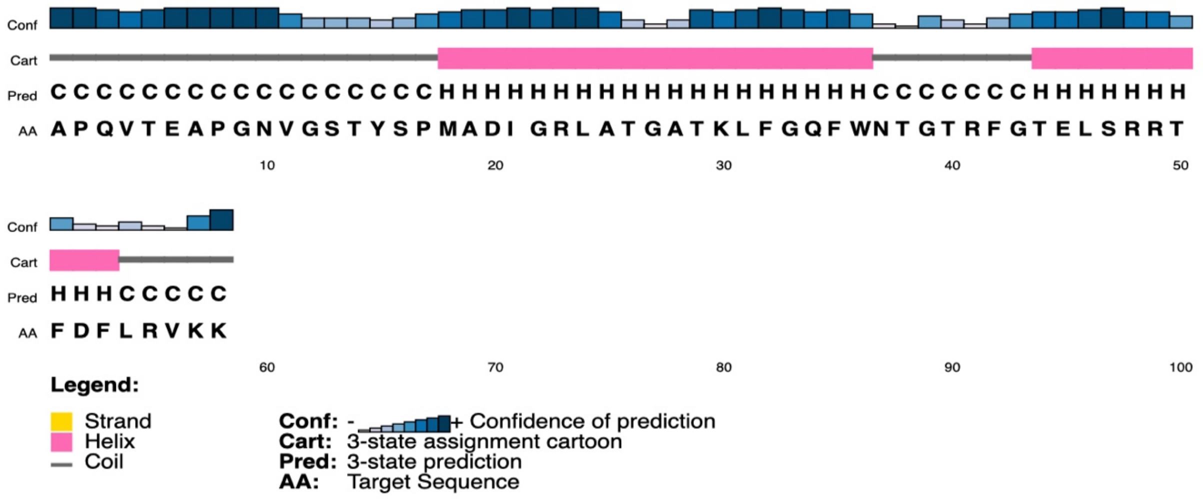

Hyp15

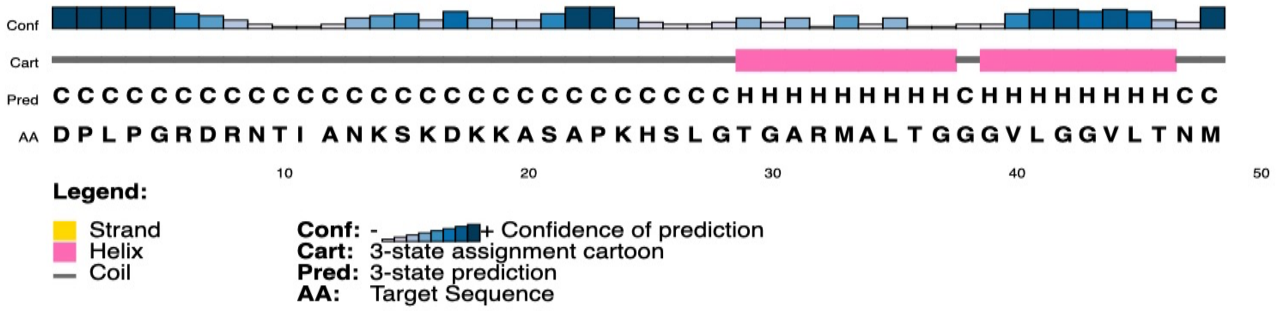

Hyp17

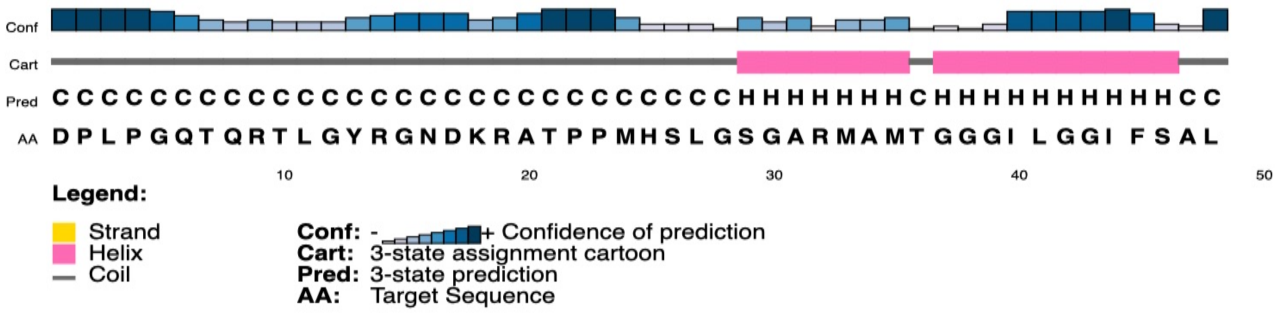

Figure S1: PSIPRED secondary structure prediction. The cartoons summarize the predictions of secondary structures of mature peptides (without SP) as calculated by PSIPRED on-line tool for hyp6.2, hyp15 and hyp17. Simple structures with one or two helixes (and no Cysteins) are predicted for the three SG+ candidates.
