# Supplementary material for: Effects of Local and Systemic Immune Challenges on the Expression of Selected Salivary Genes in the Malaria Mosquito Anopheles coluzzii
Source: Pathogens. 2021 Oct 9;10(10):1300. doi: 10.3390/pathogens10101300 (PMC8540153; doi:10.3390/pathogens10101300)
Supplement: Supplementary file 1 [file pathogens-10-01300-s001.zip › Figure_S2.pdf]

Figure S2: hyp10 and hyp12

(a)

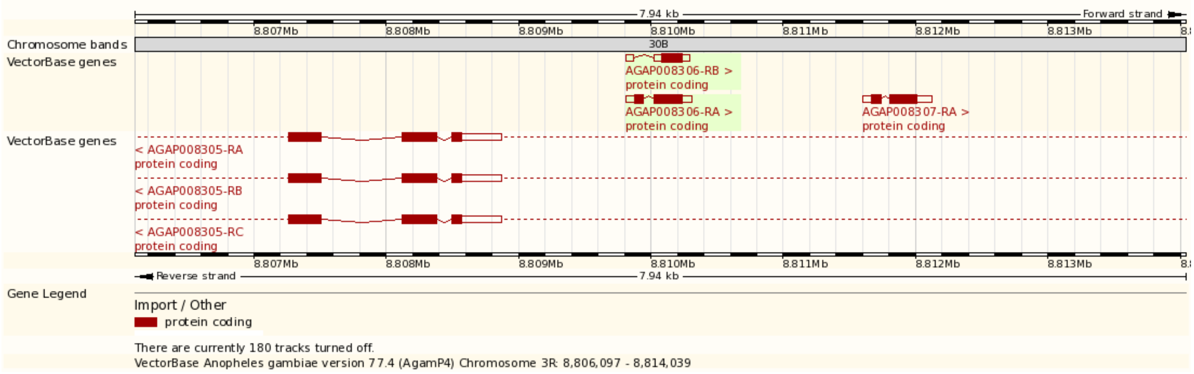

(b)

hyp10

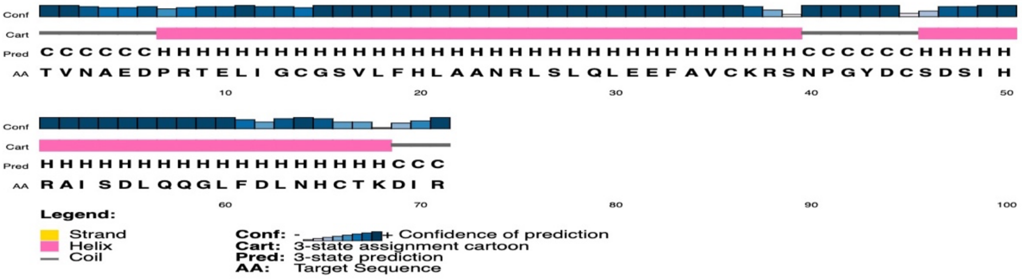

hyp12

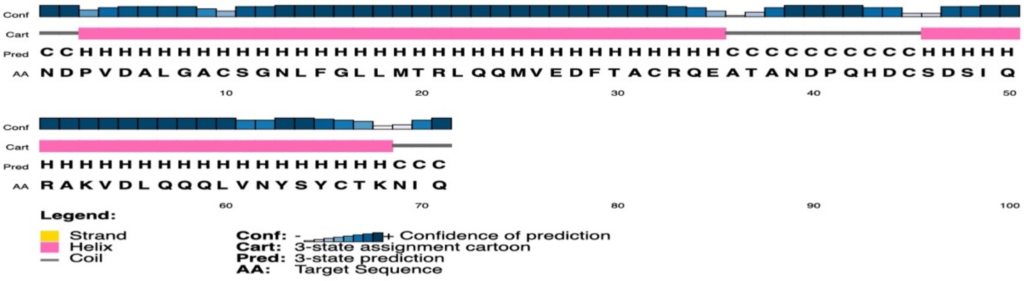

(c)

<u>Sequence alignment</u>

```
hyp12                  1 MRFCCVALIGLLLC--SVQSVTANDPVDALGACSGNLFGLLMTRLQQMVE     48
                         |||..|..:|||:.  ...:|.|.||...|..|...||.|...||...:|
hyp10                  1 MRFLTVLTVGLLVWVGVFATVNAEDPRTELIGCGSVLFHLAANRLSLQLE     50

hyp12                 49 DFTACRQEATANDPQHDCSDSIQRAKVDLQQQLVNYSYCTKNIQ     92
                         :|..|::    ::|.:||||||.||..||||.|.:.::|||:|:
hyp10                 51 EFAVCKR----SNPGYDCSDSIHRAISDLQQGLFDLNHCTKDIR     90
```

(d)

	Most frequent aminoacids		
hyp10	**14.4% Leu**	8.9% Val	
hyp12	**12% Leu**	10.9% Gln	8.7 Ala

Figure S2: hyp10 (AGAP008306)/hyp12 (AGAP008307) gene cluster. (a): scheme of the genomic region (chromosome 3R) where hyp10 and hyp12 are placed and separated by a short region of 1.2 kbp. (b): PSIPRED secondary structure prediction: the cartoons summarize the predictions of secondary structures of mature peptides (without SP) as calculated by PSIPRED on-line tool for hyp10 and hyp12. Structures with two helixes (and 4 cysteines) are predicted. (c): sequence alignment of hyp10 and hyp12: cysteines are conserved. (d): table summarizing the most frequent aminoacids in the two candidates: both candidates present a high content of leucine.
