# Supplementary material for: Effects of Local and Systemic Immune Challenges on the Expression of Selected Salivary Genes in the Malaria Mosquito Anopheles coluzzii
Source: Pathogens. 2021 Oct 9;10(10):1300. doi: 10.3390/pathogens10101300 (PMC8540153; doi:10.3390/pathogens10101300)
Supplement: Supplementary file 1 [file pathogens-10-01300-s001.zip › Figure_S3.pdf]

Figure S3: Sg2/sg2a/AGAP006505 gene cluster.

(a)

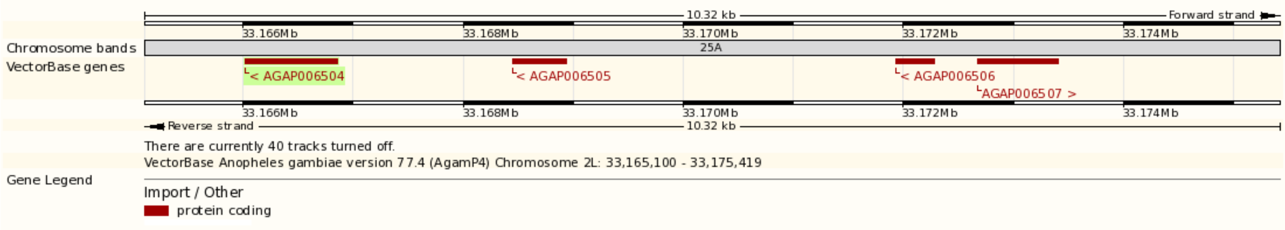

(b)

Sequence alignment

```
sg2             MKSILVTFATLSVALVVVVAIPTSFNYGGGGGYFINGTGQSFNFSGESNGTSIPGVPDFG
sg2a            MKLFLTLLLTLSV--VMVFALPAHHHSRGGDGSSANSTGQSYNNSGSPNNS--AGVPDFG
AGAP006505      MKLFLPLLSTLSV--AMVFALPAHHHSSGGEGSSANNTGHSDNYVD---SS--NSVPAIG
                ** :*  : ****  .:*.*:*: ..  ** *   * **:* *       :   .** :*

sg2             SFL----PNLGNLTQ----QFGGSSGAFPQFS-----IPSWTNFTD---------AFSSI
sg2a            FNSQSNVPGFGNGQQPGQQQQGQQGQGFPFFGQGQSGFPSFGNRQPFFGQNQQGQDG---
AGAP006505      FNPPSNVPGFGIGKQSRQQQ---QGQQFSLFTQGQGGFPNIGNDQPVFDYNQQGPYVPSA
                       * :*   *    *   ..  *  *      :*.  *

sg2             FPFFG-NVQGGGFPFFG-------------------------------------------
sg2a            -----NGQQGRGIPFFGQGGGQGGIPSFGSGQQNGGIPSLGNGQAQSGFPSFGNGQQGGN
AGAP006505      VPKYGVGQENIGPPFYFGARGDTGLPHIGYIDL-------------------------G-S
                        :  * **:

sg2             -----
sg2a            FPFFG
AGAP006505      YPIVL
```

(c)

	Most frequent aminoacids			Cysteins
sg2	**17.5% Gly**	15.8% Phe	12.3% Ser	0 Cys
sg2a	**24.3% Gly**	16.2% Gln	11.6 Phe	0 Cys
AGAP006505	**14.7% Gly**	10% Pro	10% Ser	0 Cys

Figure S3: sg2 (AGAP006504)/sg2a (AGAP006506) gene cluster. (a): scheme of the genomic region (chromosome 2L) where sg2, sg2a and AGAP006505 are placed and separated by a short region of around 8 kbp. (b): sequence alignment of sg2, sg2a and AGAP006505. (c): table summarizing the most frequent aminoacids in the sequence of the three candidates: all predicted peptides present a high content of glycine and no cysteine.
