# Supplementary material for: Effects of Local and Systemic Immune Challenges on the Expression of Selected Salivary Genes in the Malaria Mosquito Anopheles coluzzii
Source: Pathogens. 2021 Oct 9;10(10):1300. doi: 10.3390/pathogens10101300 (PMC8540153; doi:10.3390/pathogens10101300)
Supplement: Supplementary file 1 [file pathogens-10-01300-s001.zip › Figure_S4.pdf]

Figure S4: feeding assay: comparison of replicates.

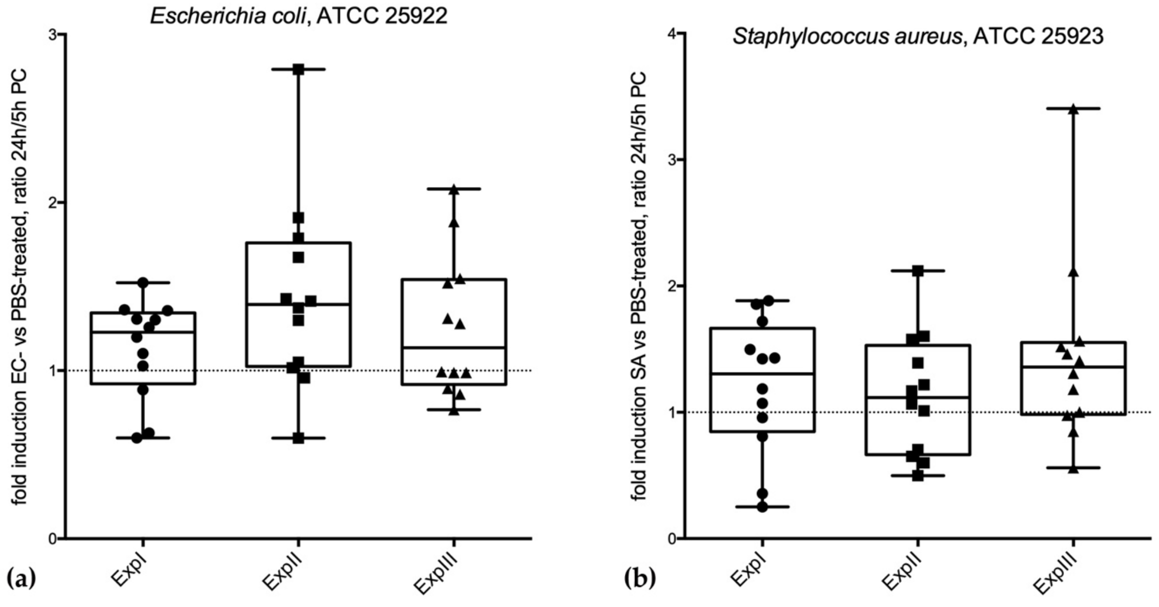

Figure S4: Feeding assay: (a) ratios of fold induction (EC-treated/PBS-treated mosquitoes) of each candidate between 5 and 24 h after challenge, in each biological replicate; (b) ratios of fold induction (SA-treated/PBS-treated mosquitoes) of each candidate between 5 and 24 h after challenge, in each biological replicate.
