# Supplementary material for: Effects of Local and Systemic Immune Challenges on the Expression of Selected Salivary Genes in the Malaria Mosquito Anopheles coluzzii
Source: Pathogens. 2021 Oct 9;10(10):1300. doi: 10.3390/pathogens10101300 (PMC8540153; doi:10.3390/pathogens10101300)
Supplement: Supplementary file 1 [file pathogens-10-01300-s001.zip › Figure_S5.pdf]

Figure S5: injection assay: candidate transcriptional modulation between 5 and 24 h post challenge.

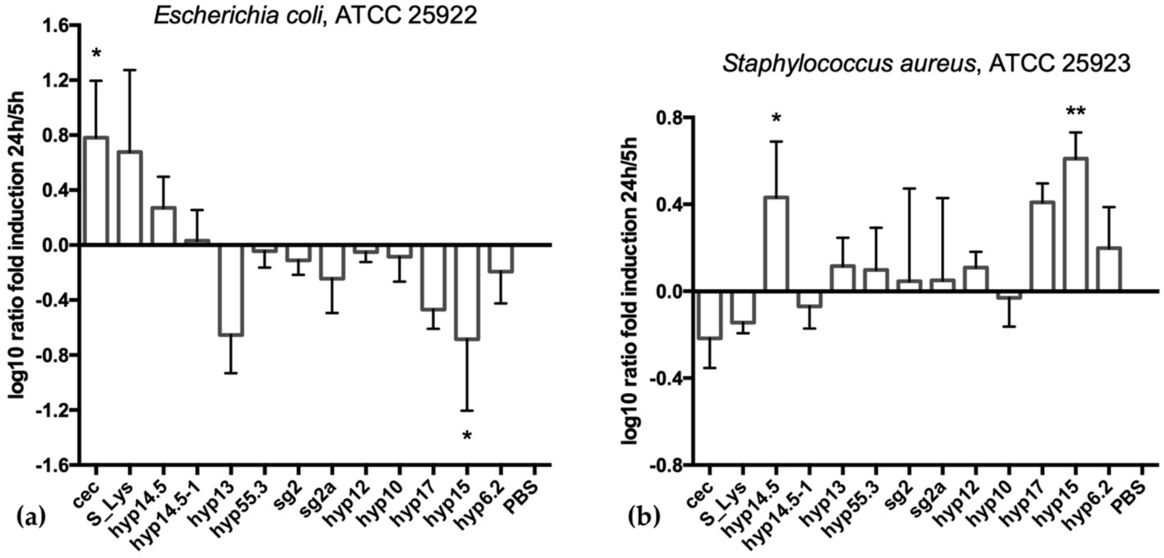

Figure S5: Injection assay: (a) log10 of fold induction (EC-treated/PBS-treated mosquitoes) of each candidate between 5 and 24 h after challenge; (b) log10 of fold induction (SA-treated/PBS-treated mosquitoes) of each candidate between 5 and 24 h after challenge. *: $p < 0.05$; **: $p < 0.01$.
